# Association of Polymorphism of Arginine-Vasopressin Receptor 1A (*AVPR1a*) Gene With Trust and Reciprocity

**DOI:** 10.3389/fnhum.2019.00230

**Published:** 2019-07-09

**Authors:** Kuniyuki Nishina, Haruto Takagishi, Hidehiko Takahashi, Masamichi Sakagami, Miho Inoue-Murayama

**Affiliations:** ^1^Graduate School of Brain Sciences, Tamagawa University, Tokyo, Japan; ^2^Brain Science Institute, Tamagawa University, Tokyo, Japan; ^3^Graduate School of Medical and Dental Sciences, Tokyo Medical and Dental University, Tokyo, Japan; ^4^Wildlife Research Center, Kyoto University, Kyoto, Japan

**Keywords:** trust game, trust, reciprocity, economic game, AVPR1A gene, gene

## Abstract

Oxytocin (OXT) is known to play an important role in trust, whereas the involvement of other peptide hormones has not been evaluated. In this study, we focused on microsatellite polymorphisms in the intron of the arginine-vasopressin receptor 1a (*AVPR1a*) gene and examined whether the association between the repeat lengths in the intron of *AVPR1a* is associated with trust and reciprocity in humans. Four-hundred and thirty-three participants played the trust game, answered the attitudinal trust question, and their buccal cells were collected. Results showed that men with a short form of *AVPR1a* tend to send more money to the opponent, even if there is a possibility of being betrayed by the opponent. Additionally, people with a short form of *AVPR1a* tended to return money to the opponent who trusts them. However, attitudinal trust was not associated with *AVPR1a*. These results indicate that arginine-vasopressin receptor 1a plays an important role in trust and reciprocal behaviors.

## Introduction

Trust is an indicator of social capital reflecting the efficiency of society, and numerous studies in the field of social science have examined human trust (Putnam et al., 1994; Fukuyama, 1995; Yamagishi, 2011). In recent years, attention has focused on the biological foundation of trust, which revealed that the peptide hormone oxytocin (OXT) synthesized in the hypothalamus regulates trust (Kosfeld et al., 2005). OXT functions in various parts of the body such as the uterus and mammary glands after transport through blood vessels from the posterior pituitary gland. OXT is axon-projected in various regions of the central nervous system, such as the striatum, amygdala, hippocampus, and others (Meyer-Lindenberg et al., 2011). Previous studies showed that OXT attenuates the stress response and enhances the reward system, as well as regulates social cognition and behavior (Domes et al., 2007; Feldman, 2017). In trust, OXT attenuates anxiety related to social risk and promotes trust by suppressing the activity of the amygdala, which is the center of emotional processing (Baumgartner et al., 2008). Additionally, because twin studies have shown that trust is inherited (Cesarini et al., 2008; Reimann et al., 2017), genetic approaches have been used to identify candidate genes of trust. Some studies showed that a polymorphism in the oxytocin receptor gene (*OXTR* rs53576) in human chromosome 3p.25.3 is associated with trust behavior and trust attitude (Krueger et al., 2012; Nishina et al., 2015), and that the amygdala volume mediates the association between *OXTR* rs53576 and trust attitude (Nishina et al., 2018). OXT is known to play an important role in trust, whereas the involvement of other peptide hormones has not been evaluated.

Arginine-vasopressin (AVP) is a peptide hormone synthesized in the hypothalamus and exerts its effects in the central nervous system (Meyer-Lindenberg et al., 2011). The AVP receptors, V1a and V1b, are distributed in the prefrontal cortex, hippocampus, amygdala, and various other regions of the brain and regulate anxiety and pair bonding behavior (Young and Wang, 2004). According to nonhuman primate studies, V1a knockout mice show low levels of anxiety (Bielsky et al., 2004) and that V1 receptor antagonist reduces anxiety-related behavior in rats (Liebsch et al., 1996). In human studies, administration of AVP increased the stress response (Shalev et al., 2011) and enhanced activation of the amygdala response to negative emotional stimuli (Brunnlieb et al., 2013). Because previous studies of OXT revealed that OXT inhibits social stress (Heinrichs et al., 2003) and attenuates activation of the amygdala response to a fearful face (Kirsch et al., 2005), OXT and AVP have opposite effects on the brain. If so, AVP inhibits trust in contrast to OXT.

The arginine-vasopressin receptor 1a (*AVPR1a*) gene is on human chromosome 12q14.2 and has two exons (Thibonnier et al., 1996). *AVPR1A* has three microsatellite polymorphisms in the promoter region, repeating two bases of (GT)_25_, a complex repeat of (CT)_4_-TT-(CT)_8_-(GT)_24_ [RS3], and a repetition of the four-nucleotide sequence GATA [RS1]. Previous studies showed that the repeat length in RS3 is associated with autism (Yirmiya et al., 2006), pair bonding behavior (Walum et al., 2008), maternal behavior (Avinun et al., 2012), and altruistic behavior in the economic game (Knafo et al., 2008; Avinun et al., 2011; Wang et al., 2016). Additionally, Meyer-Lindenberg et al. (2009) found that the repeat length in RS1 and RS3 is associated with activation of the amygdala response to emotional facial expression. Nonhuman primate studies showed that the repeat length in RS3 is associated with personality (Hopkins et al., 2012; Staes et al., 2016) and social cognition (Hopkins et al., 2014; Mahovetz et al., 2016). These results indicate that *AVPR1a* plays a role in social cognition and social behavior not only in humans but also in various other species.

Although many studies have examined microsatellite polymorphisms (RS1 and RS3) in the promoter region of *AVPR1a*, a recent study detected an association between the repeat length in the intron and personality in common marmoset. Inoue-Murayama et al. (2018) examined the association between microsatellite polymorphisms in the intron of *AVPR1a* and personality scores rated by humans for common marmoset and revealed that the short form of *AVPR1a* is associated with a high level of sociability. These results indicate that not only the repeat length in the promoter region but also that in the intron is related to sociality in nonhuman primates. However, it remains unclear whether microsatellite polymorphisms in the intron of *AVPR1a* are associated with sociality in humans. Considering the common role of *AVPR1a* in sociality with other animals, it is important to understand the evolution of human sociality.

In this study, we focused on microsatellite polymorphisms in the intron of *AVPR1a* and examined whether the association between the repeat lengths in the intron of *AVPR1a* is associated with trust and reciprocity in humans. To clarify the biological basis of trust, it is necessary to evaluate whether OXT and AVP are related to trust behavior.

## Materials and Methods

### Participants

**Figure 1:**
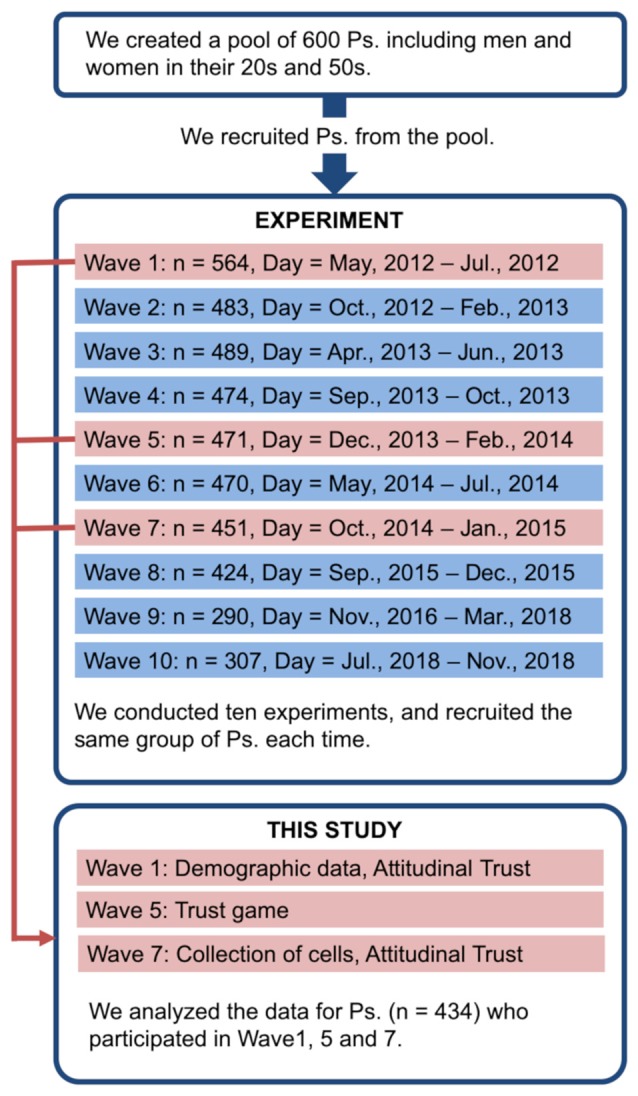
Overview of the whole research project. Ps, participants.

Six-hundred non-student residents living in Tokyo suburbs were selected from a list of 1,670 applicants who responded to a brochure distributed to approximately 180,000 households. These 600 individuals consisted of 75 men and 75 women in each 10-year age group from 20 to 59 years in the first wave (May 17, 2012). The study was conducted in ten waves for 7 years (from 2012 to 2018) and the participants repeatedly participated in the experiment. Findings concerning some of the data collected during the ten phases have been previously reported (Yamagishi et al., 2014, 2015, 2016a,b, 2017a,b; Nishina et al., 2015, 2018; Matsumoto et al., 2016). An overview of the whole research project is provided in Figure 1.

### Trust Game

The trust game was conducted in the fifth wave (December 16, 2013 to February 23, 2014). The procedures of the trust game are similar to those reported previously (Nishina et al., 2015). Participants played the trust game in a situation where anonymity was fully guaranteed. The trust game was played between pairs of participants randomly matched from among the 6–12 participants who attended the same experimental session. One member of the pair played the role of truster and the other the role of trustee. The truster was provided with JPY 1,000 by the experimenter and decided how much of these funds to transfer to the trustee in increments of JPY 100. The transferred money was then tripled and provided to the trustee. The trustee then decided how much of the tripled money to transfer back to the truster. The endowment money of JPY 1,000 was provided only to the truster, and not to the trustee. All participants were told that they would play the game twice, each time with a different partner and that their role would change. All participants played the truster role in the first game and trustee role in the second game. Trustees’ responses in the second game were measured using the strategy method. We averaged the amount of money the trustee had returned to the opponent when receiving more than 60% of the endowment and defined the behavior as reciprocity.

### Attitudinal Trust

The question of attitudinal trust was used in the first wave and in the seventh wave. Participants answered the following question, “Do you think most people would try to take advantage of you if they got a chance or would they not?” The form of answer was a binary; 0 indicated low trust and 1 indicated high trust. This question was used in two large scale social surveys: the General Social Survey and World Value Survey. We averaged the two scores of this question. The score was associated with the polymorphism of the oxytocin receptor gene (*OXTR* rs53576) reported in our previous studies (Nishina et al., 2015; Nishina et al., 2018).

### Genotyping

**Figure 2:**
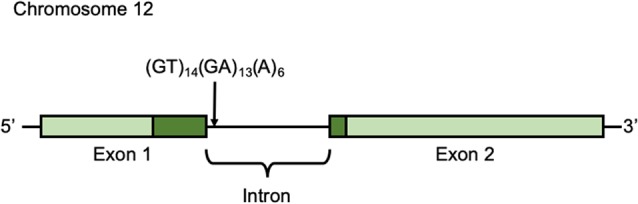
Arginine-vasopressin receptor 1a (*AVPR1a*) gene *AVPR1a* is located in chromosome 12q14.2 in humans and it has two exons. Light green shows non-coding region and Dark green shows coding region.

Participants’ buccal cells were collected in the seventh wave (October 25th, 2014 to January 25, 2015) and preserved in 90% ethanol until DNA extraction. DNA was extracted using the DNeasy Blood and Tissue Kit (QIAGEN, Hilden, Germany) according to the manufacturer’s protocol. DNA was amplified by PCR. To amplify the microsatellite polymorphism in the intron [(GT)_14_(GA)_13_(A)_6_] (Accession No. DQ177277; Figure 2), we used primers 5′-ATGTGGTCTGTCTGGGATGC-3′ (forward) and 5′-GGGTGCGACTGTAGTACACA-3′ (reverse; Inoue-Murayama et al., 2018). PCR amplification conditions were as follows: 94°C for 1 min, and then 94°C for 30 s, 60°C for 30 s, 74°C for 1 min) × 35 cycles, and final extension at 74°C for 10 min. The PCR products were analyzed with an ABI 3130*xl* DNA Sequencer and GeneMapper Software (Applied Biosystems, Foster City, CA, USA).

### Analysis

A total of 470 participants (male = 228, female = 242) played the trust game and we genotyped 449 participants (male = 221, female = 228). We analyzed 434 participants (male = 213, female = 221) for whom both behavioral and genetic data were available. Since this study was a part of the large-scale research project, we could not design a sample size suitable for this study. Instead, we report the power of analysis used in this study. The power was calculated by G*Power 3.1 software (Faul et al., 2009).

## Results

### Genotype Distribution

The distribution of the number of alleles is shown in Table 1. We defined an allele of repeat length 217 and greater than 217 as “long” (L) and that less than 217 as “short” (S). The genotype distribution of the 434 participants was 17.1% SS (*N* = 74), 50.5% SL (*N* = 219), and 32.5% LL (*N* = 141). This distribution did not significantly differ from Hardy-Weinberg equilibrium ($\chi_{\left(1\right)}^{2}$ = 0.497, *p* = 0.481). The demographic data for 434 participants are shown in Supplementary Tables S1–S5. We did not find significant differences in the proportion of sex ($\chi_{\left(2\right)}^{2}$ = 2.07, *p* = 0.356), generation ($\chi_{\left(6\right)}^{2}$ = 5.04, *p* = 0.538), education level ($\chi_{\left(2\right)}^{2}$ = 4.33, *p* = 0.115), annual income ($\chi_{\left(12\right)}^{2}$ = 7.98, *p* = 0.787), and subjective social class ($\chi_{\left(8\right)}^{2}$ = 8.95, *p* = 0.346), genotype.

**Table 1:** Frequency for *AVPR1a* genotype.

Allele	*n*	% Carriers
211	5	0.6
213	211	24.3
215	151	17.4
217	490	56.5
219	10	1.2
221	1	0.1

### Trust

**Figure 3:**
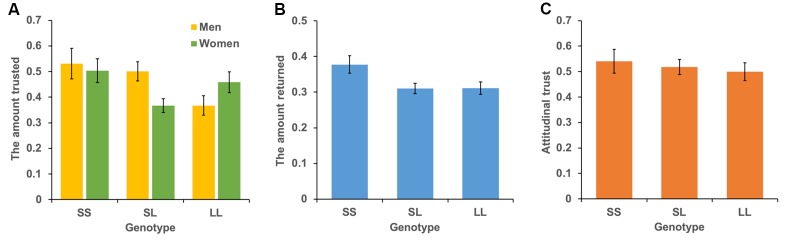
Mean levels of behavioral trust, reciprocity, and attitudinal trust for each genotype. The vertical bar represents the amount sent to the second player **(A)**, the amount returned to the first player **(B)**, and the level of attitudinal trust **(C)**. Error bars show standard error.

The mean levels of trust for the three genotypes are shown in Figure 3A. We conducted a multiple regression analysis of trust behavior. Age, sex (men = 1), dummy variable of SL genotype (= 1), and dummy variable of SS genotype (= 1) were used as independent variables. By setting the LL genotype as the baseline, this model can be used to examine the differences in trust behavior between LL vs. SL and LL vs. SS. The dependent variable is the ratio of money sent by the first player to the second player. The results showed that the SS genotype (*b* = 0.004, *SE* = 0.002, *p* = 0.015, *β* = 0.116) and age positively affected trust behavior (*b* = 0.101, *SE* = 0.048, *p* = 0.034, *β* = 0.114; model 1 in Table 2). However, the SL genotype (*b* = 0.019, *SE* = 0.036, *p* = 0.601, *β* = 0.028) and sex (*b* = 0.041, *SE* = 0.032, *p* = 0.203, *β* = 0.061) did not significantly affect trust behavior. Additionally, we examined the interaction effect of genotype and sex in model 2. The interaction effect of SL genotype and sex (*b* = 0.218, *SE* = 0.071, *p* = 0.002, *β* = 0.275) and the effect of age (*b* = 0.004, *SE* = 0.002, *p* = 0.020, *β* = 0.111) were significant. The effect of sex (*b* = −0.09, *SE* = 0.055, *p* = 0.101, *β* = −0.136), the SL genotype (*b* = −0.09, *SE* = 0.050, *p* = 0.074, *β* = −0.134) and SS genotype (*b* = 0.034, *SE* = 0.069, *p* = 0.616, *β* = 0.039), the interaction effect of SS genotype and sex (*b* = 0.128, *SE* = 0.094, *p* = 0.177, *β* = 0.109) were not significant. Since the interaction effect of the SL genotype and sex was significant, we analyze the effect of genotype for each sex. In men, the SL genotype (*b* = 0.125, *SE* = 0.054, *p* = 0.022, *β* = 0.173), the SS genotype (*b* = 0.161, *SE* = 0.070, *p* = 0.022, *β* = 0.173), and age (*b* = 0.006, *SE* = 0.002, *p* = 0.014, *β* = 0.166) positively affected trust behavior. In women, age (*b* = 0.001, *SE* = 0.001, *p* = 0.440, *β* = 0.052), the SL genotype (*b* = −0.090, *SE* = 0.046, *p* = 0.051, *β* = −0.148), and the SS genotype (*b* = 0.40, *SE* = 0.063, *p* = 0.523, *β* = 0.048) did not have an effect. The power of analysis calculated by the ΔR_2_ of tested predictors was 0.48 in model 1 and 0.87 in model 2.

**Table 2:** Results of multiple regression analysis of trust.

	Model 1				Model 2			
Variables	*b*	*SE*	*p*	*β*	*b*	*SE*	*p*	*β*
Intercept	0.239	0.069	0.001	0.000	0.315	0.073	<0.0001	0.000
Age	0.004	0.002	0.015	0.116	0.004	0.002	0.020	0.111
Sex	0.041	0.032	0.203	0.061	−0.091	0.055	0.101	−0.136
SL	0.019	0.036	0.601	0.028	−0.090	0.050	0.074	−0.134
SS	0.101	0.048	0.034	0.114	0.034	0.069	0.616	0.039
SL × Sex	-				0.218	0.071	0.002	0.275
SS × Sex	-				0.128	0.094	0.177	0.109

### Reciprocity

The mean levels of reciprocity for the three genotypes are shown in Figure 3B. We conducted the same analyses used for trust behavior analysis. The dependent variable was the ratio of money returned by the second player to the first player. The results showed that the SS genotype (*b* = 0.058, *SE* = 0.030, *p* = 0.048, *β* = 0.104) and age (*b* = 0.005, *SE* = 0.0009, *p* < 0.001, *β* = 0.241) positively affected reciprocity (model 1 in Table 3). The SL genotype (*b* = −0.005, *SE* = 0.022, *p* = 0.838, *β* = −0.011) and sex (*b* = −0.018, *SE* = 0.020, *p* = 0.369, *β* = −0.042) did not significantly affect reciprocity. Additionally, we found no significant interaction effect of genotype and sex on reciprocity (model 2 in Table 3). The power of analysis calculated by the ΔR_2_ of tested predictors was 0.52 in model 1 and 0.50 in model 2.

**Table 3:** Results of multiple regression analysis of reciprocity.

	Model 1				Model 2			
Variables	*b*	*SE*	*p*	*β*	*b*	*SE*	*p*	*β*
Intercept	0.123	0.043	0.004	0.000	0.129	0.046	0.005	0.000
Age	0.005	0.001	<0.0001	0.241	0.005	0.001	<0.0001	0.237
Sex	−0.018	0.020	0.369	−0.042	−0.023	0.035	0.514	−0.053
SL	−0.005	0.022	0.838	−0.011	−0.016	0.031	0.621	−0.037
SS	0.058	0.030	0.048	0.104	0.080	0.043	0.064	0.141
SL × Sex	-				0.023	0.044	0.599	0.046
SS × Sex	-				−0.040	0.059	0.497	−0.054

### Attitudinal Trust

The mean levels of attitudinal trust for the three genotypes are shown in Figure 3C. We conducted the same analytic model. The dependent variable was the level of attitudinal trust. We did not find an effect of SS genotype (*b* = 0.027, *SE* = 0.059, *p* = 0.647, *β* = 0.024) and SL genotype (*b* = 0.016, *SE* = 0.045, *p* = 0.725, *β* = 0.019) on attitudinal trust (model 1 in Table 4). Additionally, we did not find an interaction effect of SS genotype and sex (*b* = 0.114, *SE* = 0.119, *p* = 0.338, *β* = 0.078) and SL genotype and sex (*b* = 0.086, *SE* = 0.090, *p* = 0.336, *β* = 0.086; model 2 in Table 4).

**Table 4:** Results of multiple regression analysis of attitudinal trust.

	Model 1				Model 2			
Variables	*b*	*SE*	*p*	*β*	*b*	*SE*	*p*	*β*
Intercept	0.143	0.086	0.098	0.000	0.176	0.092	0.057	0.000
Age	0.009	0.002	<0.0001	0.213	0.009	0.002	<0.0001	0.213
Sex	0.019	0.040	0.626	0.023	−0.044	0.070	0.533	−0.052
SL	0.016	0.045	0.725	0.019	−0.028	0.063	0.658	−0.033
SS	0.027	0.059	0.647	0.024	−0.033	0.086	0.704	−0.029
SL × Sex	-				0.086	0.090	0.336	0.086
SS × Sex	-				0.114	0.119	0.338	0.078

## Discussion

Trust behavior is associated with microsatellite polymorphisms in the intron of *AVPR1a*. Men with a short form of *AVPR1a* tend to send more money to the opponent, even if there is a possibility of being betrayed by the opponent. In contrast, men with a long form of *AVPR1a* tend to keep their money. This is the first study to reveal an association between the microsatellite polymorphism in the intron of *AVPR1a* and trust behavior in humans. Our results indicate that the microsatellite polymorphism in the intron of *AVPR1a* reflects the function of arginine-vasopressin receptor 1a as well as *AVPR1a* RS1 and RS3. Previous studies showed that AVP neurons in the hypothalamus are axon-projected to the amygdala, which is the center of anxiety and fear processing (Huber et al., 2005). Additionally, AVP promotes anxiety and fearful response to emotional stimuli (Shalev et al., 2011; Brunnlieb et al., 2013). Such anxiety regulating the action of AVP can explain the results for trust behavior observed in this study. People with a long form of *AVPR1a* experience a strong effect from AVP and may be fearful of being betrayed by others. Inoue-Murayama et al. (2018) examined the association between the repeat length of the intron of *AVPR1a* and personality in the common marmoset and found that individuals with a long form of *AVPR1a* had high levels of neuroticism. These findings support the hypothesis that anxiety plays a role in trust in those with a long form of *AVPR1a*. In our previous study (Nishina et al., 2015), we found an association between the polymorphism of the oxytocin receptor gene and behavioral trust and attitudinal trust in men. A sex difference of the association of gene polymorphism and trust was also observed in the current study. These results indicate that OXT, as well as, AVP play important roles in trust in men.

Reciprocity was also associated with the repeat length of *AVPR1a*. People with a short form of *AVPR1a* tended to return money to the opponent who trusts them. The association between *AVPR1a* and reciprocity cannot be explained by the hypothesis that AVP enhances anxiety related to exploitation by others, as there is no risk of being betrayed by others in this case. AVP may not regulate the anxiety related to exploitation by others, but rather anxiety regarding the loss of money. Thus, as people with a long form of *AVPR1a* show high levels of anxiety related to the loss of money, they keep the money in either role, distrust the first player, and do not reciprocate in the second player. Huber et al. (2005) found that OXT neurons and AVP neurons differ in the location of the projection to the amygdala. This suggests that OXT and AVP regulate different types of anxiety. To evaluate this possibility, further studies are needed to examine whether *AVPR1a* is related to behavior in a trust game with a computer partner reflecting non-social risk avoidance.

AVP is related to not only anxiety but also reward processing (Meyer-Lindenberg et al., 2011). Vasopressin neurons from the hypothalamus are projected to the ventral pallidum, which forms the dopamine pathway. Avinun et al. (2011) found that individuals with the 334 allele of RS3 in *AVPR1a* showed low levels of generosity and that the 334 allele carriers maximized their self-interests through the reward system enhancement effect of AVP. However, other studies showed that AVP does not affect the motivation of maximizing self-interest, but motivation of social reward such as mutual cooperation (Rilling et al., 2012, 2014). Whether AVP affects social rewards or non-social rewards require further examination.

There were two differences between the results observed in this study and those observed in the *OXTR* study. First, we did not find an association of the polymorphism of *AVPR1a* and attitudinal trust. An important difference between behavioral trust and attitudinal trust is whether there is financial damage if the trust is betrayed. One possibility is that OXT affects attitudes like general trust by acting continuously, while AVP influences actual decision making by acting acutely depending on the situation. Another possibility is that vasopressin plays an important role in money-related decision making. Further studies are needed to examine the differences in the effects of oxytocin and vasopressin on trust. Second, while *OXTR* was related to trust but not to reciprocity (Nishina et al., 2015), *AVPR1a* was related to both trust and reciprocity. This result shows that OXT acts on trust-specific factors and that AVP acts on factors related to overall pro-social behavior. As described above, different types of anxiety may be regulated by OXT and AVP.

We found similar results as a previous study that examined the association between microsatellite polymorphisms in the intron of *AVPR1a* and sociality (Inoue-Murayama et al., 2018). In common marmoset, a short form of *AVPR1a* was related to a high level of sociality. In humans, a short form of *AVPR1a* was also related to a high level of trust and reciprocity. The common features between types of *AVPR1a* and sociality shows that the role of AVP system in sociality is an evolutionarily old issue.

We did not examine which brain function and structure mediates the association between *AVPR1a* and trust behavior. As many imaging genetic approaches are available for evaluating humans (Saito et al., 2014; Wang et al., 2016; Nishina et al., 2018), further studies are needed to determine the neural mechanism relationship between *AVPR1a* and trust behavior.

## Data Availability

The raw data supporting the conclusions of this manuscript will be made available by the authors to any qualified researcher.

## Ethics Statement

All experimental protocols were approved by the Ethics Committee of Tamagawa University, where the study was conducted, and ethics committee of Kyoto University Graduate School and Faculty of Medicine, where genotyping analysis was conducted. Each participant signed an informed consent form before the experiment.

## Author Contributions

KN, HaT, HiT, MS, and MI-M designed research. KN and HaT performed research. KN, HaT, and MI-M analyzed data and wrote the article.

## Supplementary Material

The Supplementary Material for this article can be found online at: https://www.frontiersin.org/articles/10.3389/fnhum.2019.00230/full#supplementary-material

## Conflict of Interest Statement

The authors declare that the research was conducted in the absence of any commercial or financial relationships that could be construed as a potential conflict of interest.
