# Supplementary material for: Association of Polymorphism of Arginine-Vasopressin Receptor 1A (AVPR1a) Gene With Trust and Reciprocity
Source: Front Hum Neurosci. 2019 Jul 9;13:230. doi: 10.3389/fnhum.2019.00230 (PMC6630777; doi:10.3389/fnhum.2019.00230)
Supplement: Supplementary file 1 [file Table_1.DOCX]

Table S1 Genotype distribution by sex

| Sex | Genotype | | |
| --- | --- | --- | --- |
|  | SS | SL | LL |
| Men | 39 | 100 | 74 |
| Women | 35 | 119 | 67 |
