# Supplementary material for: Association of Polymorphism of Arginine-Vasopressin Receptor 1A (AVPR1a) Gene With Trust and Reciprocity
Source: Front Hum Neurosci. 2019 Jul 9;13:230. doi: 10.3389/fnhum.2019.00230 (PMC6630777; doi:10.3389/fnhum.2019.00230)
Supplement: Supplementary file 2 [file Table_2.DOCX]

Table S2 Genotype distribution by generation

| Generation | Genotype | | |
| --- | --- | --- | --- |
|  | SS | SL | LL |
| 20s | 13 | 38 | 29 |
| 30s | 16 | 64 | 37 |
| 40s | 25 | 52 | 41 |
| 50s | 20 | 65 | 34 |
