# Supplementary material for: Association of Polymorphism of Arginine-Vasopressin Receptor 1A (AVPR1a) Gene With Trust and Reciprocity
Source: Front Hum Neurosci. 2019 Jul 9;13:230. doi: 10.3389/fnhum.2019.00230 (PMC6630777; doi:10.3389/fnhum.2019.00230)
Supplement: Supplementary file 3 [file Table_3.DOCX]

Table S3 Genotype distribution by education

| Education | Genotype | | |
| --- | --- | --- | --- |
|  | SS | SL | LL |
| Less than college | 15 | 72 | 40 |
| College+ | 59 | 147 | 101 |
