# Supplementary material for: Association of Polymorphism of Arginine-Vasopressin Receptor 1A (AVPR1a) Gene With Trust and Reciprocity
Source: Front Hum Neurosci. 2019 Jul 9;13:230. doi: 10.3389/fnhum.2019.00230 (PMC6630777; doi:10.3389/fnhum.2019.00230)
Supplement: Supplementary file 4 [file Table_4.DOCX]

Table S4 Genotype distribution by annual income

| Annual income | Genotype | | |
| --- | --- | --- | --- |
|  | SS | SL | LL |
| 0 | 5 | 22 | 13 |
| ~150M | 22 | 47 | 35 |
| ~300M | 8 | 43 | 29 |
| ~500M | 17 | 46 | 26 |
| ~700M | 13 | 35 | 17 |
| ~1000M | 7 | 9 | 14 |
| +1000M | 1 | 6 | 5 |

Note. Four participants did not answer annual income.
