# Supplementary material for: Association of Polymorphism of Arginine-Vasopressin Receptor 1A (AVPR1a) Gene With Trust and Reciprocity
Source: Front Hum Neurosci. 2019 Jul 9;13:230. doi: 10.3389/fnhum.2019.00230 (PMC6630777; doi:10.3389/fnhum.2019.00230)
Supplement: Supplementary file 5 [file Table_5.DOCX]

Table S5 Genotype distribution by subjective social class

| Subjective social class | Genotype | | |
| --- | --- | --- | --- |
|  | SS | SL | LL |
| L-Lower | 4 | 6 | 10 |
| U-Lower | 13 | 45 | 25 |
| L-Middle | 42 | 103 | 72 |
| U-Middle | 15 | 63 | 34 |
| Upper | 0 | 2 | 0 |
